# Supplementary material for: Heat adaptation of phage T7 under an extended genetic code
Source: Virus Evol. 2021 Dec 1;7(2):veab100. doi: 10.1093/ve/veab100 (PMC8923235; doi:10.1093/ve/veab100)
Supplement: veab100_Supp [file veab100_supp.zip › Sequencing_Analysis.zip!/Sequencing_Analysis/S2/summary.html]

BRESEQ :: Summary Statistics


|  |  |
| --- | --- |
|  | ***breseq***  version 0.30.1    mutation predictions \| marginal predictions \| summary statistics \| genome diff \| command line log |

## Read File Information

|  | read file | reads | bases | passed filters | average | longest | mapped |
| --- | --- | --- | --- | --- | --- | --- | --- |
| errors | S2\_52\_S17\_L001\_R1\_001 | 829,512 | 249,683,112 | 100.0% | 301.0 bases | 301 bases | 82.9% |
|  | **total** | **829,512** | **249,683,112** | 100.0% | 301.0 bases | **301** bases | 82.9% |

## Reference Sequence Information

|  |  | seq id | length | fit mean | fit dispersion | % mapped reads | description |
| --- | --- | --- | --- | --- | --- | --- | --- |
| coverage | distribution | NC\_001604.1 | 39,937 | 5366.1 | 89.3 | 100.0% | na |
|  |  | **total** | **39,937** |  |  | 100.0% |  |

**fit dispersion** is the ratio of the variance to the mean for the negative binomial fit. It is =1 for Poisson and >1 for over-dispersed data.

## New Junction Evidence

### Junction Candidates Tested

| option | limit | actual |
| --- | --- | --- |
| Number of alignment pairs examined for constructing junction candidates | ≤ 100000 | 8765 |
| Coverage evenness (position-hash) score of junction candidates | ≥ 2 | ≥ 2 |
| Test this many junction candidates (n). May be smaller if not enough passed the coverage evenness threshold | 100 ≤ n ≤ 5000 | 42 |
| Total length of all junction candidates (factor times the reference genome length) | ≤ 0.1 | 0.635 |

### Junction Skew Score Calculation

| reference sequence | pr(no read start) |
| --- | --- |
| NC\_001604.1 | 0.31958 |

**pr(no read start)** is the probability that there will not be an aligned read whose first base matches a given position on a given strand.

### Final Junction Predictions

| option | value |
| --- | --- |
| Coverage evenness (position-hash) score of predicted junctions must be | ≥ 3 |
| Skew score of predicted junction (−log10 probability of unusual coverage evenness) must be | ≤ 3 |
| Number of bases that at least one read must overlap each uniquely aligned side of a predicted junction | ≥ 1 |

## Read Alignment Evidence

| option | value |
| --- | --- |
| Mode | Consensus/Mixed Base |
| Ploidy | 1 (haploid) |
| Consensus mutation E-value cutoff | 10 |
| Consensus frequency cutoff | 0.8 |
| Consensus minimum coverage each strand | OFF |
| Polymorphism E-value cutoff | 10 |
| Polymorphism frequency cutoff | 0.2 |
| Polymorphism minimum coverage each strand | OFF |
| Polymorphism bias cutoff | OFF |
| Predict indel polymorphisms | YES |
| Skip indel polymorphisms in homopolymers runs of | OFF |
| Skip base substitutions when they create a homopolymer flanked on each side by | OFF |

## Software Versions

| program | version |
| --- | --- |
| bowtie2 | 2.2.6 |
| R | 3.4.0 |

## Execution Times

| step | start | end | elapsed |
| --- | --- | --- | --- |
| Read and reference sequence file input | 12:03:51 07 Aug 2017 | 12:04:08 07 Aug 2017 | 17 seconds |
| Read alignment to reference genome | 12:04:08 07 Aug 2017 | 12:11:24 07 Aug 2017 | 7 minutes 16 seconds |
| Preprocessing alignments for candidate junction identification | 12:11:24 07 Aug 2017 | 12:11:45 07 Aug 2017 | 21 seconds |
| Preliminary analysis of coverage distribution | 12:11:45 07 Aug 2017 | 12:12:40 07 Aug 2017 | 55 seconds |
| Identifying junction candidates | 12:12:40 07 Aug 2017 | 12:12:42 07 Aug 2017 | 2 seconds |
| Re-alignment to junction candidates | 12:12:42 07 Aug 2017 | 12:16:20 07 Aug 2017 | 3 minutes 38 seconds |
| Resolving alignments with junction candidates | 12:16:20 07 Aug 2017 | 12:17:04 07 Aug 2017 | 44 seconds |
| Creating BAM files | 12:17:04 07 Aug 2017 | 12:17:46 07 Aug 2017 | 42 seconds |
| Tabulating error counts | 12:17:46 07 Aug 2017 | 12:18:17 07 Aug 2017 | 31 seconds |
| Re-calibrating base error rates | 12:18:17 07 Aug 2017 | 12:18:19 07 Aug 2017 | 2 seconds |
| Examining read alignment evidence | 12:18:19 07 Aug 2017 | 12:24:13 07 Aug 2017 | 5 minutes 54 seconds |
| Polymorphism statistics | 12:24:13 07 Aug 2017 | 12:24:15 07 Aug 2017 | 2 seconds |
| Output | 12:24:15 07 Aug 2017 | 12:25:23 07 Aug 2017 | 1 minute 8 seconds |
| **Total** | | | **21 minutes 32 seconds** |
